# Syphilis among HIV-positive men who have sex with men in Ghana: The 2023 biobehavioral survey

**DOI:** 10.1371/journal.pone.0310909

**Published:** 2024-09-25

**Authors:** Chris Guure, Adikwor Ewoenam Puplampu, Samuel Dery, Gamji Rabiu Abu-Ba’are, Seth Kwaku Afagbedzi, Stephen Ayisi Addo, Kwasi Torpey

**Affiliations:** 1 Department of Biostatistics, School of Public Health, University of Ghana, Accra, Ghana; 2 School of Nursing, University of Rochester, Rochester, New York, United States of America; 3 National AIDS/STI Control Programme, Ghana Health Service, Accra, Ghana; 4 Department of Population, Family and Reproductive Health, School of Public Health, University of Ghana, Accra, Ghana; Taipei Veterans General Hospital, TAIWAN

## Abstract

**Background:**

Apart from HIV acquisition, men who have sex with men are at a higher risk of sexually transmitted infections, especially syphilis. Although the intersection between syphilis and HIV poses a formidable challenge among key populations who are known to be vulnerable to many health threats, there is little known about syphilis infection among MSM living with HIV in Ghana. This study seeks to investigate the burden of syphilis and address the existing knowledge gap by exploring behavioral, healthcare access, and structural factors influencing the syphilis burden within the HIV-positive MSM population.

**Method:**

This study was conducted in 2023 as part of the bio-behavioral survey (BBS) among men who have sex with men (MSM) in Ghana. A cross-sectional survey that used a respondent-driven sampling (RDS) approach was conducted in the old ten regions of Ghana. Data was collected on 3,420 participants, however, 857 HIV-positive MSM were included in this study since it focused on syphilis among HIV-positive MSM in Ghana. The study estimated the prevalence of syphilis among MSM living with HIV and provided a 95% confidence interval across different categories of explanatory variables. Bivariate and multivariable logistic regression models were used to identify factors associated with overall syphilis prevalence. All other analyses were weighted due to the complex design of the study.

**Results:**

The overall prevalence of syphilis was 23.83% (95% CI: 20.44, 27.58). HIV-positive men who only had sex with men had a 29.77% (95% CI: 23.90, 36.40) prevalence of syphilis compared to a prevalence of 9.50% (95% CI: 2.56, 29.53) recorded by HIV-positive MSM who were attracted to mostly females. Participants who ever had receptive anal sex recorded a higher prevalence 26.79% (95% CI: 22.78, 31.23) than those who never had receptive anal sex 12.86% (95% CI: 8.02, 19.99). The odds of syphilis among HIV-positive MSM who never used condoms with regular sex partners in the last 6 months were 41.08 (aOR: 41.08 at 95% CI 1.24–136.42; p = 0.038).

**Conclusion:**

There is a high prevalence of syphilis among HIV-positive men who have sex with men in Ghana, especially among those who engage in unprotected anal sex and have multiple sexual partners. Our findings underscore the dire need for targeted interventions to address the dual brunt of HIV and syphilis among the MSM population in Ghana.

## Introduction

Globally, the Human Immunodeficiency Virus (HIV) epidemic continues to be a persistent public health issue, and Ghana, like many other nations, has grappled with its impact on various aspects of life [1–4]. Although the estimated worldwide median HIV prevalence among adults aged 15–49 years is 0.7%, this prevalence is highest among transgenders (10.3%) and among men who have sex with men (MSM) (7.7%).

In addition to HIV acquisition, men who have sex with men are considered to be at a higher risk of sexually transmitted infections, especially syphilis [5]. The intersection of infectious diseases poses a formidable challenge, particularly within key populations that are known to be vulnerable to multiple health threats [6,7]. Among these populations, MSM stands out as a subpopulation facing compounded and heightened risk of both HIV and syphilis due to less frequent use of condoms during anal sex [8–12]. The CDC 2022 reported a 25% increase in no-condom use during sex among HIV-positive MSM from an estimated 46% to about 71% within 5 years (2012 to 2017). The concurrent presence of syphilis and HIV in HIV-positive MSM can lead to significant health challenges [13,14]. Syphilis has the potential to increase HIV viral load and lower CD4 T-cell counts in individuals with HIV, thereby heightening the risk of transmitting HIV to their partners [13,15].

Although the World Health Organization has set global targets to reduce new cases of syphilis by 90% in 2030, its prevalence continues to increase among HIV-positive men who have sex with men [16–18]. The global response to syphilis reduction has been slow and far from achieving WHO targets [19]. The narrative is no different in Ghana, where, like other developing countries, syphilis infection is higher among HIV-positive men who have sex with men [17,20]. In the Ghanaian population, the cumulative prevalence of Syphilis infections among the 4,181 blood donors during the five years (2012–2016) was estimated at 2.58%, and 0.24% from sentinel sites in Ho [21]. The prevalence of 4.5% was also reported by [22] for the general population of Accra. However, the [20] revealed that the prevalence of syphilis among MSM in Ghana was 1% per the total male population.

Untreated syphilis can spread to the brain and nervous system, ears, and eyes, causing neurological, eye, and ear problems and even death. However, it is curable. To control syphilis, the greatest challenge is to prevent transmission [23,24].

In Ghana, approximately 52.9% of women accessing antenatal care services were tested for syphilis at any visit out of which 2.8% of them had positive syphilis serology. This reinforces the need for our study to investigate the factors contributing to the high prevalence of syphilis among HIV-positive MSM in Ghana [1,25], because homosexual behavior in the country is compounded by the intense stigma and criminalization [26] and this environment of fear and discrimination exacerbates the risk of syphilis transmission, as MSM may avoid seeking care or disclosing their sexual behaviors to healthcare providers [27]. The convergence of these two infectious diseases raises critical public health concerns in the country. The co-occurrence of syphilis and HIV is of particular concern, as each of the infections can exacerbate the severity and transmission of the other [19]. Designing effective prevention and intervention strategies tailored to the specific needs of HIV-positive MSM with an increased prevalence of syphilis in Ghana is crucial. Yet little is known about its prevalence in Ghana and other West African countries. This study aims to fill the existing knowledge gap by investigating the social, behavioral, and healthcare-related factors that contribute to the burden of syphilis in the HIV-positive MSM population. By doing so, we hope to gain valuable insights that will aid in the efforts to improve 1) the health and well-being of MSM communities in the face of dual infection risks in Ghana and 2) national policies for MSM in Ghana.

## Methods

### Survey design and settings

The study was part of a 2023 bio-behavioral survey (BBS) among MSM and TGs in Ghana. A cross-sectional survey was conducted using a Respondent Driven Sampling (RDS) approach. The research was carried out in Ghana’s 10 former regions, namely Western, Central, Eastern, Greater Accra, Volta, Ashanti, Brong Ahafo, Northern, Upper East, and Upper West. In each region, a team of 5 to 7 individuals was selected (these are key population members who met the eligibility criteria, identified through formative assessment or nominated by stakeholders and MSM community leaders, well-connected within their social networks, well-regarded by their peers, supportive of the survey’s goals, in separate social networks and not members of CBOs or NGOs), trained, and given 3 coded recruitment coupons each. These individual seeds were asked to give these coupons to persons within their social network. Potential respondents who were recruited by the seeds were screened for eligibility and enrolled if they met the survey inclusion criteria and further consented to participate. After participating in the survey, these individuals are also given recruitment coupons and asked to distribute them to their peers. This process continues until the target sample size and survey parameters are achieved. Each cycle of recruitment and participation adds an additional sampling wave and effectively created recruitment chains. The number of recruits per person was restricted to 3 to facilitate long chains which helps the recruitment of diverse social networks. Participants were uniquely identified using a fingerprint scanner and unique code to avoid duplicates.

### Ethical considerations

This study was approved by two institutions: the Ghana Health Service Ethics Review Committee (GHS-ERC:007/01/22) and the Noguchi Memorial Institute of Medical Research (NMIMR-IRB CPN 028/21-22). All participants in this study were required to sign an informed consent form. The study was voluntary, and the consenting process was explained to participants so that they would willingly decide to participate. Participants had the flexibility to withdraw at any time during the survey process.

### Inclusion and exclusion criteria

Participants were included if the person was biologically born a male, aged 18 years and above, self-reported consensual anal sex with another man within the last 12 months, live, work, or socialize in any of the regions in Ghana, can communicate in English or any Ghanaian language, and can provide informed consent to survey participation. However, potential participants who could not provide informed consent for any reason, including being under the influence of drugs or alcohol, were excluded from the survey.

### Sample size

The sample size (3,420) was calculated with an expected change in the indicator of consistent condom use (48.2%) among MSM and based on detecting a change of 15 percentage points with an alpha of 5% and 80% power. A design effect of 1.5 was factored in with a non-response rate of 10%. The final sample size after adjusting for strata (10) amounted to 3,420 for the entire country. However, since this study focused on syphilis among HIV-positive men who have sex with men, a total of 857 HIV-positive MSM were included in this analysis.

### Data collection and management

The study utilized a structured questionnaire designed based on WHO, Joint United Nations Programme on HIV/AIDS, Family Health International 360, Centers for Disease Control and Prevention, and U.S. President’s Emergency Plan for AIDS Relief bio-behavioral survey guidelines for the population at risk of HIV as well as the previous Integrated Biological and Behavioral Surveillance Survey (IBBSS) for men who have sex with men (Ghana Men study) questionnaire and Ghana Demographic Health Survey tools [28]. Trained interviewers conducted face-to-face interviews with the respondents using a mobile data collection software called Research and Electronic Data Capture (REDCap). The survey started on the 5th of May 2023 and ended on the 14th of July 2023 across all the regions of Ghana.

### Biological testing

Counselling for HIV and syphilis was done in a private setting by trained counsellors. HIV and syphilis tests were conducted on-site by trained survey staff in line with national HIV testing and counselling guidelines and established survey-specific protocols. Trained laboratory staff collected 6mls of blood from consenting participants. Universal precautions were observed, and blood samples were labelled with the same unique participant identification number as the behavioural survey. Tests for all participants were carried out on-site and they were counselled before and after. Those willing to take their results received them immediately. Those who tested positive for HIV and/or syphilis were linked by the site counsellor to be enrolled in an appropriate treatment facility for treatment if not already enrolled elsewhere.

#### HIV

HIV testing was conducted on-site with whole blood using the First Response HIV 1+2 / Syphilis Combo Card Test. This rapid, qualitative screening, in vitro diagnostic test, detects antibodies specific to HIV (type 1 & 2) and *Treponema pallidum*. Reactive specimens for HIV were confirmed further with OraQuick test kits. A third test using SD Bioline confirmed a positive result [29].

#### Syphilis infection

Tests for *Treponema pallidum* (TP) antibodies were conducted on-site with the First Response HIV 1+2 / Syphilis Combo Card Test on site. TP antibody positives were re-tested on-site using the Abbot ADK Syphilis 3.0 to determine infections.

### Study variables

#### Outcome variable

Syphilis results among MSM who tested positive for HIV. The variable was coded as dichotomized, with “1” representing HIV-positive MSM who tested positive for syphilis and “0” if HIV-positive MSM did not test positive for syphilis.

#### Independent variables

Independent variables used for this study were age, marital status, region, sexual orientation, sex attracted to the most, ever had receptive anal sex with a man, number of men had receptive anal sex within the last six months, condom use during last sex (receptive/ insertive). Others included frequency of condom use with regular sex partners in the last six months, ever been given free condoms through an outreach service, drop-in center, or health center, use of free distributed condoms, number of sexual partners in the last six months, ever received money or goods for sex, knows where to go for sexual health or STI check-ups. We also obtained and included in the analysis MSM who have been in contact with any peer educator in the last 12 months, ever tested for HIV, know where to go if they want to take an HIV test to know their status, MSM who had ever told anyone that they were MSM community members, felt ashamed because of being a community member in the last 12 months and thinking less of oneself because of being a community member.

### Statistical analysis

The statistical analyses were performed using Stata SE version 17 (Stata Corp). Descriptive statistics were conducted using weighted frequencies and percentages for categorical variables and mean and standard deviations for continuous variables to describe the socio-demographic characteristics, sexual behaviors, and healthcare access of the study participants. Also, due to the complex design of the MSM data, all other analyses performed were weighted and accounted for stratification and design effect in Stata. The estimates of syphilis prevalence among HIV-positive MSM were provided with a 95% confidence interval across the various categories of explanatory variables. Bivariate and multivariable logistic regression models were conducted to assess factors associated with syphilis prevalence among the study population. A simple bivariate logistic regression model was employed to extract variables that were significantly associated with the outcome at a 95% confidence level and subsequently, a multivariable logistic regression model was fitted using age as a priori and included all the significant variables. Variables with p-values greater than 20% in the multivariable model were excluded from the final model.

## Results

### Characteristics of study population

From the data set using the weighted frequencies and percentages, a total of 857 HIV-positive men who had sex with men were identified, with a median age of 25(IQR 23–29) years. A higher proportion (70.75%) of participants were young adults between 20–29 years, and 3.37% were older and above 40 years. Most of the MSM live in the Greater Accra region (27.75%). Over 90% were single or never married. A higher proportion (80.92%) ever had receptive anal sex with a man, and 38.85% had experienced it with more than 3 men. About 40% did not use condoms during the last insertive/ receptive anal sex. Participants who had more than 4 sexual partners were more (37.63%) than those who had two (20.93%) and one (27.52%). A proportion of 21.99% had experienced stigma and discrimination for being MSM. An estimated 11.44% and 2.43% have a few times and often, respectively, thoughtless of themselves because they are community members (Table 1).

**Table 1:** Characteristics of study participants and prevalence of syphilis among HIV-positive men who have sex with men in Ghana.

	Total N = 857 n(%)	Prevalence of syphilis [95% CI]	p-value
**Overall**	857(100)	23.83 [20.44, 27.58]	
**Age (m, IQR)**	25 (23, 29)		
**Age group (n = 857)**			0.200
< = 19	40(4.80)	21.25[9.81, 40.08]	
20–29	587(70.75)	24.65[20.56, 29.25]	
30–39	175(21.09)	26.11[18.55, 35.42]	
40+	28(3.37)	5.28[1.26, 19.56]	
Non-response	30		
**Marital status**			
Single, never married	751(90.89)	24.57[20.91, 28.63]	0.298
Married/cohabiting	61(7.41)	15.62[7.53, 29.62]	
Separated/divorced	10(1.18)	17.39[4.32, 49.58]	
Widowed	4(0.52)	63.12[10.34, 96.21]	
Non-response	31		
**Region name**			<0.001
Greater Accra	238(27.75)	40.23[32.63, 48.32]	
Ashanti	146(17.06)	17.67[11.81, 25.58]	
Volta	86(10.06)	23.22[13.55, 36.85]	
Western	99(11.61)	13.37[7.03, 23.97]	
Eastern	127(14.87)	21.30[13.91, 31.19]	
Central	60(7.02)	27.15[15.93, 42.28]	
Brong Ahafo	74(8.67)	7.41[3.01, 17.10]	
Northern	23(2.69)	1.54[0.21, 10.53]	
Upper East	0(0.00)		
Upper West	2(0.28)	2.89[0.34, 20.78]	
**Sexual orientation**			0.045
Gay/Homosexual	442(54.57)	27.98[23.00, 33.56]	
Bisexual	360(44.44)	19.47[14.96, 24.95]	
Transgender	8(0.99)	39.83[14.11, 72.74]	
Non-response	47	15.58[6.57, 32.64]	
**Sex attracted to the most**			0.048
Only male	342(41.40)	29.77[23.90, 36.40]	
Mostly male	284(34.38)	21.98[16.93, 28.01]	
Equally male and female	172(20.82)	18.75[12.31, 27.50]	
Mostly female	28(3.40)	9.50[2.56, 29.53]	
Non-response	31		
**Ever had receptive anal sex with a man**			0.005
Yes	670(80.92)	26.79[22.78, 31.23]	
No	158(19.08)	12.86[8.02, 19.99]	
Non-response	30		
**Number of men had receptive anal sex with in the past 6 months (n = 679)**			0.025
None	71(10.40)	33.29[19.84, 50.15]	
1 man	216(31.79)	17.78[12.26, 25.08]	
2 men	129(18.95)	26.06[18.39, 35.53]	
3+men	264(38.85)	32.79[26.05, 40.33]	
**Condom use during last sex (Receptive/Insertive)**			<0.000
No condom use	253(40.48)	17.09[12.31, 23.24]	
Used condom	372(59.52)	20.67[16.18, 26.02]	
Non-response	232		
**Frequency of condom used with regular sex partners in the last 6 months (n = 756)**			0.121
Always	221(55.25)	23.86[17.45, 31.74]	
Sometimes	176(44.00)	29.48[21.60, 38.81]	
Never	3(0.75)	78.67[32.61, 96.57]	
Non-response	356	23.41[18.34, 29.37]	
**Ever been given free condoms through an outreach service, drop-in center or health center**			
No	271(32.97)	16.30[11.63, 22.37]	0.006
Yes	551(67.03)	28.01[23.55, 32.96]	
Non-response	35		
**Use free distributed condoms**			
No	195(23.84)	16.06[10.68, 23.42]	0.025
Yes	623(76.16)	26.81[22.67, 31.40]	
Non-response	39		
**Number of sexual partners in the last 6 months (n = 828)**		0.003
1 partner	228(27.52)	20.24[14.81, 27.04]	
2 partners	173(20.93)	16.53[10.54, 24.98]	
3 partners	115(13.92)	20.14[13.01, 29.84]	
4+ partners	312(37.63)	32.66[26.34, 39.69]	
**Ever received money or goods for sex**			
No	410(49.88)	20.00[15.75, 25.06]	0.028
Yes	412(50.12)	28.56[23.33, 34.44]	
Non-response	35		
**Knows where to go for sexual health or STI check-up**			0.046
No	90(10.91)	13.86[8.13, 22.62]	
Yes	735(89.09	25.48[21.69, 29.67]	
Non-response	32		
**Been in contact with any peer educator in the last 12 months**			0.024
No	266(32.20)	17.72[13.04, 23.61]	
Yes	560(67.80)	27.27[13.04, 23.61]	
Non-response	32		
**Ever tested for HIV**			0.011
No	94(11.01)	10.65[5.62, 19.26]	
Yes	730(85.20)	25.85[22.05, 30.05]	
**Do you know where you can go if you want to take an HIV test to know your status**			0.006
No	47(5.70)	5.31[1.98, 13.49]	
Yes	778(94.30)	25.31[21.66, 29.34]	
Non-response	32		
**Ever told anyone that you are an MSM community member**			0.026
No	415(50.30)	19.76[15.63, 24.66]	
Yes	410(49.70)	28.69[23.40, 34.64]	
Non-response	32		
**I have felt ashamed because I am a community member in the last 12 months**			**0.043**
Never	652(79.22)	22.41[18.65, 26.68]	
Once	46(5.59)	30.67[15.59, 51.45]	
A few times	81(9.84)	33.29[22.47, 46.21]	
Often	20(2.43)	46.71[24.23, 70.60]	
Does not apply because no one knows me	24(2.92)	8.11[2.58, 22.74]	
Non-response	34		
**In the last 12 months, I have thought less of myself because I am a community member (n = 826)**			0.050
Never	649(78.95)	23.43[19.54, 27.83]	
Once	29(3.53)	23.94[10.98, 44.54]	
A few times	94(11.44)	28.97[19.27, 41.06]	
Often	20(2.43)	59.05[37.07, 77.92]	
Does not apply because no one knows me	30(3.65)	6.40[2.09, 17.95]	
Non-response	4		

### Prevalence of syphilis among HIV-positive men who have sex with men in Ghana

The overall prevalence of syphilis among HIV-positive MSM in Ghana was 23.83% (95% CI: 20.44, 27.58). Stratifying them into regions, Greater Accra recorded the highest prevalence, 40.23% (95% CI: 32.63, 48.32), followed by the Central region at 27.15% (95% CI: 15.93, 42.28), with Upper West at 2.89% (95% CI: 0.34, 20.78) being the least. HIV-positive MSM who were attracted to only males had 29.77% (95% CI: 23.90, 36.40) prevalence of syphilis; however, those who were attracted to mostly females recorded a lesser prevalence of 9.50% (95% CI: 2.56, 29.53). Participants who ever had receptive anal sex recorded a higher prevalence, 26.79% (95% CI: 22.78, 31.23), than those who never had receptive anal sex, 12.86% (95% CI: 8.02, 19.99). About 27.27% (95% CI: 13.04, 23.61) of those who have been in contact with any peer educator in the last 12 months, 25.85% (95% CI: 22.05, 30.05) ever tested for HIV, 28.69% (23.40, 34.64) ever told anyone that they were community members were diagnosed with syphilis. Participants who had 4 or more sexual partners had a higher prevalence of 32.66% (95% CI: 26.34, 39.69) than 2 sexual partners at 16.53% (95% CI: 10.54, 24.98) (Table 1).

### Factors associated with syphilis among HIV-positive men who have sex with men in Ghana

In the adjusted model MSM from Greater Accra (aOR: 19.40 at 95% CI 5.23–72.03; p<0.000), Ashanti (aOR: 4.88 at 95% CI 1.06–22.39; p = 0.042), Volta (aOR: 5.75 at 95% 1.30–25.50; p = 0.021) and Central regions (aOR: 17.45 at 95% CI 3.62–84.03; p<0.000) were significantly more likely to be diagnosed with syphilis compared to participants in Brong Ahafo Region. The odds of syphilis among HIV-positive MSM who never used condoms with regular sex partners in the last 6 months were 41.08 (aOR: 41.08 at 95% CI 1.24–136.42; p = 0.038) more likely to have syphilis than participants who always used condoms. MSM who had 2 partners were 65% (aOR: 0.35 at 95% CI: 0.15–0.86; p = 0.022) less likely to be diagnosed with syphilis than participants with 4 or more partners. HIV-positive MSM who have often thought less of themselves because they are community members had 18.49 (aOR: 18.49 at 95% CI: 2.91–117.41; p = 0.002) higher odds of being diagnosed with syphilis than those that never did (Table 2).

**Table 2:** Factors associated with syphilis among HIV-positive men who have sex with men in Ghana.

	Unadjusted odds ratio		Adjusted odds ratio	
	cOR[95% CI]	p-value	aOR[95% CI]	p-value
**Age group (n = 829)**				
< = 19	0.82[0.32, 2.11]	0.687	0.86[0.24, 3.09]	0.811
20–29	1.00[reference]		1.00[reference]	
30–39	1.08[0.66, 1.78]	0.761	1.50[0.70, 3.20]	0.297
40+	0.17[0.04, 0.76]	0.02	0.10[0.01, 1.40]	0.087
**Marital status**				
Single, never married	1.00[reference]		-	
Married/cohabiting	0.57[0.24, 1.33]	0.191	-	-
Separated/divorced	0.65[0.14, 3.06]	0.582	-	-
Widowed	5.26[0.35, 78.67]	0.229	-	-
**Region name**				
Greater Accra	8.41 [3.09, 22.95]	<0.000	19.40 [5.23, 72.03]	<0.000
Ashanti	2.68[0.93, 7.73]	0.068	4.88[1.06, 22.39]	0.042
Volta	3.78 [1.19, 11.98]	0.024	5.75[1.30, 25.50]	0.021
Western	1.93[0.59, 6.32]	0.277	4.19[0.91, 19.21]	0.065
Eastern	3.38 [1.15, 9.96]	0.027	3.24[0.72, 14.60]	0.126
Central	4.66 [1.45, 14.92]	0.010	17.45 [3.62, 84.03]	<0.000
Brong Ahafo	1.00 [reference]		1.00 [reference]	
Northern	0.20[0.02, 0.82]	0.151	-	-
Upper East	-	-	-	-
Upper West	0.37[0.03, 3.99]	0.414	-	-
**Sexual orientation**				
Gay/Homosexual/Lesbian	1.00[reference]			
Bisexual	0.62[0.41, 0.94]	0.024	-	-
Transgender	1.70[0.41, 7.04]	0.461	-	-
**Sex attracted to the most**				
Only male	1.00[reference]		1.00[reference]	
Mostly male	0.66[0.43, 1.03]	0.069	0.47[0.23, 0.93]	0.030
Equally male and female	0.55[0.30, 0.97]	0.040	0.37[0.16, 0.89]	0.027
Mostly female	0.25[0.06, 1.02]	0.053	0.38[0.05, 2.65]	0.327
**Ever had receptive anal sex with a man**			
No	1.00[reference]			1.00[reference]
Yes	2.48[1.40, 4.38]	0.002	2.02[0.81, 5.04]	0.132
**Number of men had receptive anal sex within the past 6 months**			
None	1.02[0.47, 2.22]	0.955	-	-
1 man	0.44[0.26, 0.76]	0.003	-	-
2 men	0.72[0.42, 1.26]	0.249	-	-
3+men	1.00[reference]			
**Condom use during last sex (Receptive/Insertive)**			
No	1.00[reference]			
Yes	1.26[0.78, 2.06]	0.346	-	-
**Frequency of condom use with regular sex partners in the last 6 months**		
Always	1.00[reference]		1.00[reference]	
Sometimes	1.33[0.75, 2.37]	0.326	1.20 [0.62, 2.32]	0.579
Never	11.77[1.48, 93.57]	0.020	41.08 [1.24, 136.42]	0.038
**Ever been given free condoms through an outreach service, drop-in center or health center**				
No	1.00[reference]		-	-
Yes	2.00[1.27, 3.15]	0.003	-	-
**Use free distributed condoms**				
No	1.00[reference]		-	
Yes	1.91[1.14, 3.22]	0.014	-	-
**Number of sexual partners in the last 6 months**				
1 partner	0.52[0.32, 0.85]	0.009	1.11[0.48, 2.55]	0.807
2 partners	0.41[0.22, 0.75]	0.004	0.35[0.15, 0.86]	0.022
3 partners	0.52[0.28, 0.95]	0.034	0.67[0.25, 1.81]	0.431
4+ partners	1.00[reference]		1.00[reference]	
**Ever received money or goods for sex**			
No	1.00[reference]			-
Yes	1.60[1.07, 2.38]	0.021	-	-
**Knows where to go for sexual health or STI check-up**				
No	0.47[0.25, 0.89]	0.02	-	-
Yes	1.00[reference]		-	
**Been in contact with any peer educator in the last 12 months**				
No	0.57[0.37, 0.89]	0.012	-	-
Yes	1.00[reference]		-	
**Ever tested for HIV**				
No	0.34[0.17, 0.71]	0.004	-	-
Yes	1.00[reference]		-	
**Do you know where you can go if you want to take an HIV test to know your status**				
No	0.17[0.6, 0.47]	0.001	0.05[0.01, 0.31]	0.002
Yes	1.00[reference]		1.00[reference]	
**Ever told anyone that you are an MSM community member**			
No	1.00[reference]			-
Yes	1.63[1.10, 2.43]	0.015	-	-
**I have felt ashamed because I am a community member in the last 12 months**				
Never	1.00[reference]		-	
Once	1.53[0.62, 3.78]	0.355	-	-
A few times	1.73[0.96, 3.12]	0.07	-	-
Often	3.03[1.08, 1.04]	0.035	-	-
Does not apply because no one knows me	0.31[0.09, 1.04]	0.058		
**In the last 12 months, I have thought less of myself because I am a community member**				
Never	1.00[reference]		1.00[reference]	
Once	1.03[0.39, 2.70]	0.955	0.95[0.24, 3.73]	0.939
A few times	1.33[0.74, 2.39]	0.334	0.88[0.32, 2.46]	0.814
Often	4.71[1.87, 11.88	0.001	18.49[2.91, 117.41]	0.002
Does not apply because no one knows me	0.22[0.07, 0.73]	0.013	-	-

## Discussion

This study contributes to the growing body of literature on the MSM community, specifically addressing the prevalence of syphilis infection among HIV-positive men who have sex with men in Ghana. We assessed syphilis infection in this key population and identified its predictors among 857 participants who were included in this study. Our findings revealed that the prevalence of syphilis among HIV-positive MSM was 23.83%, indicating that a considerable proportion of HIV-positive MSM in Ghana are co-infected with syphilis, showing a high existence of co-occurrence of these infections. This finding is higher than a study conducted by Mashingaidze et al. 2023, [30] in South Africa, which recorded a prevalence of 5.2%. However, a systematic review and meta-analysis by Wu et al. 2021, [5] presented the prevalence rates across different countries, and although some countries Nanjing, China (30.9%), Thailand (33%) recorded higher prevalence of syphilis among HIV-positive MSM, others America (1.1%), China (14.9%), Ecuador (4.8%), UK (1.9%), India (8.4%-14%), Indonesia (4.3%) recorded lower prevalence than that of Ghana [13,15,31–36]. Studies conducted on the general population in Cape Coast, Ghana noted a syphilis prevalence of 8.5% [27], 16.5% among inmates in prisons and 7.9% among prison officers [37] among others [21,22]. This prevalence in the general Ghanaian population is lower, compared to the findings from this study. Ghana’s high prevalence highlights the role of higher-risk sexual behaviors in contributing to the transmission of STIs among MSM communities, and by extension, to the general population through MSM who may be bisexual [38]. The WHO 2024, [39] reported that, globally, syphilis is highly prevalent in the MSM population, with even higher rates among MSM living with HIV. Similarly, in the United States, men who have sex with men continue to represent the majority of syphilis cases; most are co-infected with HIV [40,41]. Our study found that nearly half of HIV-positive MSM are bisexual, with a syphilis prevalence of 19.47%. This suggests that the MSM community may inadvertently increase the risk of syphilis transmission to their female partners, as well as to fetuses and newborns. A report by the WHO 2016, [16] showed that over 300,000 fetal and neonatal deaths, along with nearly 250,000 additional infants at an increased risk of early death, were caused by exposure to the bacterium that causes syphilis.

Additionally, this study revealed that substantial regional differences exist in the prevalence of syphilis among MSM groups in Ghana which correlates with previous findings on both HIV and syphilis in the general population [42]. This indicates that the risk of syphilis infection varies across regions in Ghana, highlighting the need for targeted regional interventions to address both HIV and syphilis among MSM communities, rather than relying solely on national strategies. The intersectionality of HIV and syphilis among MSM community across the various regions underscores the complex dynamics of sexual health within this population and emphasizes the urgent need for comprehensive prevention and control strategies [43]. More so, a significant number of MSM experienced feelings of shame and low self-esteem due to their sexual orientation and identity within the MSM community in Ghana. These feelings are likely exacerbated by internalized homophobia, social stigma and discrimination, all of which negatively impact mental health. Low self-worth and shame are common psychological stressors that can lead to severe mental health problems such as anxiety, depression and suicidal ideation, especially when individuals feel unsupported or isolated. This finding is consistent with other studies [7,44–46]. Policymakers and health professionals must develop targeted interventions that will address the unique needs of the MSM communities in the country.

Furthermore, the odds of contracting syphilis among HIV-positive MSM were higher among those who do not use condoms (unprotected anal sex), have four or more partners (multiple sexual partners), and are attracted to only males. This is consistent with similar research done on risky sexual behavior among key populations in different countries [19,17,47,48]. Other studies confirmed that the availability of antiretroviral therapy has lowered the fear of HIV transmission in both HIV-infected and uninfected men who have sex with men, contributing to inconsistent condom use and indulgence in multiple sexual partners, which has indirectly increased the incidence of syphilis [49–51]. Interventions aimed at reducing high-risk sexual behaviors and promoting safer sex practices are critical in palliating the spread of both HIV and syphilis among the MSM population in Ghana.

### Recommendation

Consistent and correct condom use should be encouraged during sexual encounters through targeted educational campaigns that highlights the importance of practicing condom use in preventing the transmission of HIV and STIs. Additionally, comprehensive education on risk reduction strategies, such as reducing the number of sexual partners and encouraging people to make informed decisions about their sexual health, should be emphasized. Regular screening and treatment of STIs should be advocated. Stakeholders and governmental agencies should develop targeted interventions that consider the unique cultural, social, and economic factors that may influence sexual behavior and access to healthcare services in these regions. Moreover, there should be ongoing research and surveillance efforts to monitor trends in syphilis prevalence and associated risk factors among HIV-positive MSM. This data can inform the development and refinement of targeted interventions and public health strategies.

## Study limitations/Challenges

1. All eligible and consented participants were given compensation (transportation reimbursement) of 100 Ghana cedis, this encouraged some MSM to try to enroll more than once but most if not all were identified by the biometric system that was used. There were other people who, due to the compensation, pretended to be MSM and came to the venues to be enrolled. Though a very strict screening procedure was used, we acknowledge the possibility of a few non-MSM taking part in the survey.
2. There is the possibility that some participants felt shy and as a consequence might have provided socially desirable responses.
3. Stigma and discrimination could have had an influence on the enrollment process at some of the venues.
4. Though efforts were made by the team and community advisory group to select seeds across the spectrum (different towns and cities), the social structure of the networks might not have conformed strictly to the assumptions required by the RDS approach.

## Conclusion

There is a high prevalence of syphilis among HIV-positive men who have sex with men in Ghana, especially among those who engage in unprotected anal sex and have multiple sexual partners. Our findings underscore the dire need for targeted interventions to address the dual brunt of HIV and syphilis among the MSM population in Ghana. This is because HIV co-infection with *Treponema pallidum* has been associated with increased HIV viral load and decreased CD4 counts [40]. Integrated and comprehensive strategies focusing on promoting safer sex practices, combating stigma and discrimination, and improving access to healthcare services in reducing the prevalence of syphilis and improving sexual health outcomes of HIV-positive MSM in Ghana are needed.
